# Evaluation of the Potential of Collagen from Codfish Skin as a Biomaterial for Biomedical Applications

**DOI:** 10.3390/md16120495

**Published:** 2018-12-08

**Authors:** Ana M. Carvalho, Alexandra P. Marques, Tiago H. Silva, Rui L. Reis

**Affiliations:** 1 3B’s Research Group, I3Bs–Research Institute on Biomaterials, Biodegradables and Biomimetics, University of Minho, Headquarters of the European Institute of Excellence in Tissue Engineering and Regenerative Medicine Avepark–Parque de Ciência e Tecnologia, Zona Industrial da Gandra, 4805-017 Barco, Guimarães, Portugal; ammpfc@i3bs.uminho.pt (A.M.C.); apmarques@i3bs.uminho.pt (A.P.M.); rgreis@i3bs.uminho.pt (R.L.R.); 2 ICVS/3B’s–PT Government Associate Laboratory, 4805-017 Braga/Guimarães, Portugal; 3 The Discoveries Centre for Regenerative and Precision Medicine, Headquarters at University of Minho, Avepark, 4805-017 Barco, Guimarães, Portugal

**Keywords:** marine-origin collagen, codfish, biophysical characterization, biologic activity, ASTM guidelines, biomedical application, marine biomaterials

## Abstract

Collagen is one of the most widely used biomaterials, not only due its biocompatibility, biodegradability and weak antigenic potential, but also due to its role in the structure and function of tissues. Searching for alternative collagen sources, the aim of this study was to extract collagen from the skin of codfish, previously obtained as a by-product of fish industrial plants, and characterize it regarding its use as a biomaterial for biomedical application, according to American Society for Testing and Materials (ASTM) Guidelines. Collagen type I with a high degree of purity was obtained through acid-extraction, as confirmed by colorimetric assays, SDS-PAGE and amino acid composition. Thermal analysis revealed a denaturing temperature around 16 °C. Moreover, collagen showed a concentration-dependent effect in metabolism and on cell adhesion of lung fibroblast MRC-5 cells. In conclusion, this study shows that collagen can be obtained from marine-origin sources, while preserving its bioactivity, supporting its use in biomedical applications.

## 1. Introduction

Collagen is the most abundant protein in vertebrates, playing a dominant role in the maintenance of the biological and structural integrity of tissues, and contributing to the molecular architecture, shape, and mechanical features [1]. It is a trimeric molecule consisting of three polypeptide α-chains, which are woven in a triple helix forming homotrimers or heterotrimers, depending on the collagen type. Currently, about 28 types of collagen are identified in human tissues. About one-half of the total human body collagen is present in the skin, being mostly collagen type I [2,3], with each tropocollagen (individualized) molecule composed of two equal α_1_ chains and one α_2_ chain.

Collagen has revealed excellent biocompatibility, biodegradability and biorenewal, and weak antigenicity [4]. Moreover, collagen-cell interaction is well understood. Such properties and knowledge led to the development of a plethora of collagen-based biomedical devices, including drug delivery systems, surgical sutures, hemostatic agents and tissue-engineering applications [2,5]. In addition, collagen has a well-preserved structure and amino acid composition between species, which contributes to the mentioned properties when considering the use of non-human collagens in the biomedical context.

Considering commercial exploitation, the available collagen is mainly obtained from terrestrial animals, namely bovine, porcine, caprine and rat species. Despite the fine screening of transmissible diseases, the risk of transmission of bovine spongiform encephalopathy, ovine and caprine scrape, foot-and-mouth disease and swine influenza from animals to humans is nonetheless a concern [6,7]. Collagen obtained from fish may be a safer alternative, since there is no known risk of disease transmission. Moreover, fish processing by-products, such as skin, bones and scales, are abundantly available, assuring the sustainable exploitation of marine resources through the valorization of residues, while addressing associated environmental pollution issues [8,9]. Besides fish, many other marine organisms from different taxa have been studied as origins of biomass for the extraction of collagen, from a fundamental perspective or as raw-material for biopolymer production. Examples can be found on marine sponges [10,11,12,13,14,15], jellyfish [16,17,18], anemones [19,20], corals [21], squids [22,23,24] and different echinoderms [25,26,27].

To allow the use of such marine-origin collagen in pharmaceutical, cosmetics or biomedical fields, adequate processing of raw material is required, following highly demanding and strict regulations, which can be particularly challenging when using fish by-products as skins and hence associated with high cost. Although those efforts would be fully covered by the high added value of the envisaged applications, one should also consider the expected differences between marine origin collagens and mammal collagens, particularly regarding denaturation temperature, which is commonly lower for marine collagens [9], but also the rheological properties exhibited by aqueous solutions of this molecule, which we can infer in a rough way by the normally less-viscous character of marine collagen solutions or dispersions when compared with those produced with mammal collagens. These differences are commonly associated with alterations in the hydroxyproline contents and/or hydroxylation degree of proline amino acids, which are reduced in marine collagens. This has been recently confirmed by a comparative study by Bao et al. [28], which also raised the possibility of a synergistic effect of hydroxyproline and cysteine, affecting not only the thermal properties of collagen, but also its mechanical properties. Other possibilities to overcome the limited mechanical properties may be the establishment of extraction protocols that render more native macromolecular entities, namely, preserving interaction with glycosaminoglycans [29], or addressing processing features, namely, chemical crosslinking [30,31].

Fish collagen has been obtained from different species, including rohu, catla, megrim, dover sole, codfish, hake, carp, shark, spotted golden goatfish, tuna, niger triggerfish, tilapia and northern pike [6,32,33,34,35,36,37,38,39,40,41]. In these studies, the main concern is the establishment of valuable extraction processes, and collagen physical and chemical properties, while the biomedical potential is rarely addressed, with reports on biological assessment or biological validation of the extracted collagen typically lacking. This study is intended not only to explore codfish skin as a sustainable and valuable source of collagen, by establishing an extraction methodology to produce high-purity collagen extracts from industrial by-products, but also to address its biomedical potential by characterization according to the guidelines established by the appropriate American Society for Testing and Materials (ASTM) Standard F 2212 [42]—a document designed by ASTM International that defines the methodologies to be used as a reference for the characterization of collagen entitled for use as a component of surgical implants and tissue-engineered medical products, including the use of the extracted codfish collagen as a substrate for human fibroblast cell culture.

## 2. Results and Discussion

### 2.1. Electrophoretic Profiling of Acid-Soluble Collagen Extracted from Codfish Skin

**Figure 1:**
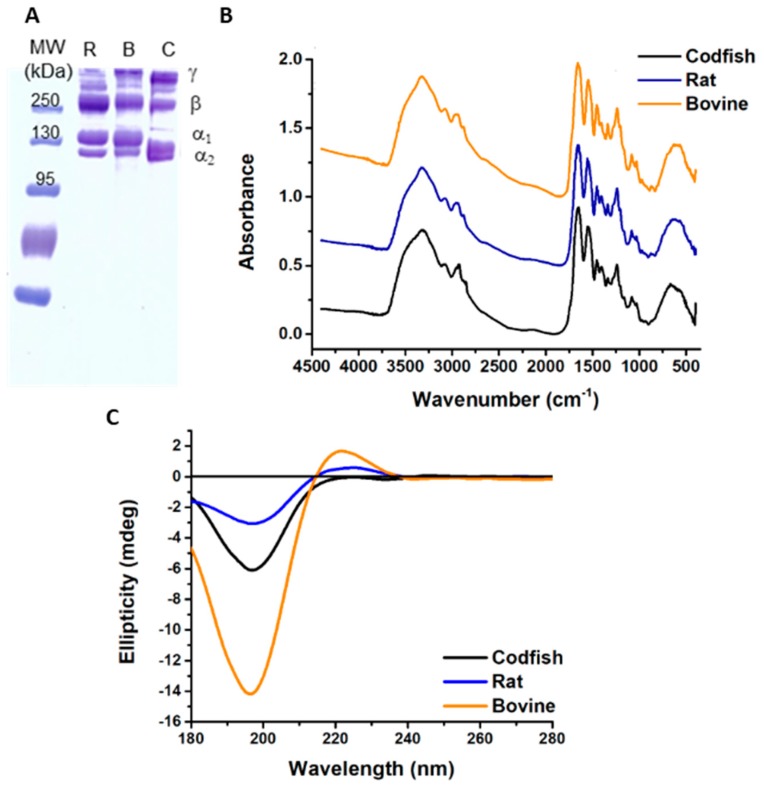
Characterization of collagen extracted from skin of codfish (C) and rat (R) and bovine (B) collagen type I. (**A**) Sodium dodecyl sulfate-polyacrylamide electrophoresis evidences the molecular structure and organization of collagen type I by the presence of monomer ((α_1_)_2_(α_2_)); dimers (β) and trimers (γ) components are also present. Moreover, similar molecular weights (MW) between the collagens were found. (**B**) Fourier transform infrared spectra of collagens exhibits the main vibrations of collagen molecular organization, amide A, amide B, amide I, amide II and amide III. (**C**) Circular dichroism spectra show a positive band with maximum ellipticity at 225 nm, confirming the presence of triple-helix structure in collagens.

To confirm the type of collagen extracted and its purity, SDS-PAGE was performed, which destroyed the structure of the protein and separated the respective components according to their electrophoretic mobility (Figure 1A). Extracted collagen has two clear bands attributed to two different kinds of α-chains, as observed in the case of collagen type I from both rat and bovine species [43]. However, both α-chains of collagen from codfish presented a higher electrophoretic mobility, attributed to lower molecular weights. The molecular weight of α_1_ was about 130 kDa for mammal (rat and bovine) collagens and 120 kDa for codfish collagen, while α_2_ was about 115 kDa for mammal and 110 kDa for codfish collagen. Skierka et al. studied the extraction processes of collagen from codfish, including skin and backbone [44,45]. Despite the differences in the extraction methodology used and source of collagen between the studies, it was possible to obtain collagen type I with a similar molecular weight as reported. The differences observed in molecular weight compared to mammalian collagen are usually associated with differences in the amino acid composition. The two α_1_ chains (α_1I_ and α_1II_) could not be distinguished because only one band is observed under the electrophoretic conditions employed, as both chains are equal and thus have the same electrophoretic migration pattern [46,47]. Nevertheless, a ratio of α_1_/α_2_ equal to 2 was observed, indicating that the α_1_ band corresponds to double the quantity of chains detected in the α_2_ band, which suggests that extracted collagen is type I [43]. In all samples, intramolecular cross-linking β- and γ-components were detected: γ-components were richer in collagen obtained from skin of codfish than in the rat or bovine collagens, while β-components were found in lower amount in the former. Intensive intra- and intermolecular covalent crosslink takes place universally to stabilize the collagen fibrils against proteolytic degradation and promotes the desired tensile strength to the stroma [3]. However, the crosslink of adjacent chains into dimers (β-chains) or trimers (γ-chains), may inhibit the proper folding of collagen protein to a triple-helix conformation, as it may interfere in the degree of freedom of individual monomers. The purification of α-monomers from the collagen may represent an advantage when a triple helix conformation is required.

### 2.2. Fourier-Transformed Infrared (FTIR) Spectroscopy Analysis

The FTIR spectra of collagen extracted from codfish skin and commercial collagen type I from rat tail and bovine skin are exhibited in Figure 1B. All three spectra share great similarities. They exhibit several features characteristic of collagen molecular organization: amino acids linked together by peptide bounds give rise to infrared active vibration modes amide A and B and amide I, II and III. The characteristic infrared absorption of amide A, associated with N-H stretching vibration, occurred between 3400 and 3440 cm^−1^; the respective absorption peaks of collagen extracted from codfish, and collagen type I extracted from rat tail and from bovine skin were found at 3329, 3320 and 3324 cm^−1^, respectively. A shift in the amide A absorption band to lower frequencies appears when a peptide is involved in hydrogen bonds [48,49,50]. Moreover, band symmetry suggests that a low amount of water is present [51]. Amide B peaks codfish, rat and bovine collagen, representing asymmetrical stretch of CH_2_, were found at 2877, 2879 and 2878 cm^−1^, respectively [52]. The amide I bond is usually observed in the range 1600–1700 cm^−1^ and corresponds to the stretching vibration of C=O along the polypeptide backbone of proteins, which is a sensitive area to protein secondary structure, and therefore used in analysis [48,50,52]. The amide I band in codfish and rat collagen was found at 1649 cm^−1^, while from bovine collagen was found at 1656 cm^−1^. The amide II band refers to N-H bending vibration coupled with C-N stretching vibrations and is commonly found at the 1550–1600 cm^−1^ range [51,53]. In codfish and rat collagen, the amide II band was found at 1554 cm^−1^ and from bovine collagen was found at 1546 cm^−1^. The shift to lower frequencies observed on the band of bovine collagen when compared with collagen obtained from codfish and rat reflects the existence of hydrogen bonds. Amide III bands were found at 1239 cm^−1^ in all samples, reflecting complex intermolecular interactions, including C-N stretching and N-H in-plane bending from amide linkages, as well as absorptions from wagging vibrations from CH_2_ groups, from the glycine backbone and proline side-chains [51]. Moreover, the ratio between amide III and the band at 1450 cm^−1^ was close to 1, which is an indication of the presence of the triple helix structure of collagen in powder [49,54]. The similarities between collagen obtained from codfish and commercial collagens suggested that their structures are quite similar in a dry state [55]. The presence of nucleic acids, phospholipids and lipids was identified in all samples at about 1030, 1228 and 1454 cm^−1^, respectively [52].

### 2.3. Extract Purity

Total protein concentration was assessed using a micro BCA kit, while collagen content was quantified using Sircol assay and SDS-PAGE. The protein content of codfish extract was 87.7 ± 3.6%, whereas the collagen content was 75 ± 20%. Syrius red, the dye in the Sircol assay, binds specifically to hydroxyproline in collagen. This amino acid residue is found in marine-origin collagen in lower amounts than in mammalian-origin collagen, which is a standard calibrator for the assay. Therefore, collagen content determined by Sircol assay is not accurate, due to the difference in hydroxyproline contents between both collagen origins, with a higher purity degree expected than that determined [56]. By SDS-PAGE imaging analysis using Image J, collagen purity was determined to be around 90%, in line with the purity found in rat and bovine collagen type I, thus showing it to be a more appropriate methodology to address the purity of marine collagen extracts.

### 2.4. Circular Dichroism (CD)

It is important to assess whether the extracted collagen type I is in the native–triple helical structure, or in the denatured form–coil structure. Collagen is known to adopt polyproline II-like helical conformation, which is characterized by CD with a positive maximum absorption band at around 222 nm. Herein, the solutions of collagen from codfish skin, rat tail and bovine skin were prepared at 0.1 mg/mL and analysed for conformation using the CD spectroscopy in the far-UV region (Figure 1C). CD spectra of bovine collagen show a preeminent positive band at 222 nm, which is typical of collagen’s triple-helical structure, and is also visible, although with less intensity, for rat tail collagen; on the other hand, codfish collagen did not exhibit a clear positive band at 222 nm, which may give an indication of at least partial denaturation of the protein upon extraction. Intermolecular interactions of triple helix are disassociated by dilute acids and by repulsion forces that occur between the same charges of collagen monomers [5]. Moreover, the presence of intramolecular interactions in collagen is highly dependent on the amino acid composition, namely, on the presence of proline and hydroxyproline due to the stereochemical contribution of the pyrrolidine ring and the extra hydrogen bonds that may be formed [57,58].

### 2.5. Amino Acid Composition

Collagen type I consists of 20 different amino acids organized as three α-chains which wrap around each other forming a characteristic triple-helix conformation. This structure is stabilized by the presence of glycine residue in every three residues, high content of proline (Pro) and hydroxyproline (OHyp), interchain hydrogen bonds, and electrostatic interactions involving lysine and aspartate [59,60]. The amino acid composition of collagen is presented in Table 1. In all samples, glycine was the most abundant amino acid, with 266–333/1000 residues. Glycine content is the principal feature that affects the triple helix formation [61] and accounts for more than 25% of all amino acids in all samples, which is in good agreement with the presence of Gly in each third amino acid residue. This percentage was smaller in the marine-origin collagen. Alanine, proline, hydroxyproline and glutamine were the most common amino acids found in the spiral structure, which agrees with their reported high content [62]. In particular, OHyp and Pro contents in collagen obtained from codfish skin were 39 and 62/1000 residues, respectively. The degree of hydroxylation of proline was calculated to be 46.8%, 46.6% and 38.7% for rat, bovine and codfish collagens, respectively and is correlated with triple helix stability, and thus with the denaturing temperature of the protein [41]. While proline and hydroxyproline were found in lower amounts than in mammalian collagen, serine and methionine were present in higher amounts in fish collagen. Such findings are in agreement with those observed for collagens extracted from other marine-species sources [6,7,35,36,37,38,39,41,49,52,54].

### 2.6. Denaturation Temperature

The structural level of collagen organization in solution depends on the ratio of triple helix:coil structures. Collagen is denatured when 90% of molecules are in coil state and un-denatured when at least 70% of molecules keep their triple helical structure [5].

The denaturation temperature of the collagens solubilized in 20 mM acetic acid was assessed by monitoring the viscosity of those collagen solutions at different temperatures (Table 2). Denaturation temperatures were calculated from the thermal denaturation curve as 18.25 ± 0.40 °C, 35.34 ± 0.03 °C and 40.08 ± 0.01 °C for codfish, bovine and rat origins, respectively. Moreover, 70% of the molecules kept their native structure until 15.77 ± 0.09 °C, 33.19 ± 0.04 °C and 39.65 ± 0.02 °C, respectively. This is in agreement with the tendency suggested by the amino acid content results, where lower amounts of Pro and OHpro are associated with lower denaturing temperature [41].

### 2.7. Heavy Metals

Metals and other elements can be naturally present in fishes. Mercury (Hg), lead (Pb), cadmium (Cd), and arsenic (As) are of concern due to accumulation in tissues. The content of heavy metal impurities in codfish collagen was determined considering permissible limits. All the elements studied were below the detection limits of the equipment, as follows: Pb < 2.5 ppm; Cd < 0.25 ppm; Hg < 0.5 ppm; and As < 0.35 ppm.

The U.S. Pharmacopeia Convention (USP) specifies limits for the amounts of elemental impurities in drug products, including the amount of elemental impurities present in daily dose (permitted daily exposure based on a 50 Kg person) and in large volume parenteral (LVP—daily dose greater than 100 mL). Concentration limits for parenteral drug products with a maximum dose of 10 g/day are Cd 0.25 ppm, Pb 0.5 ppm, As 0.15 ppm, and Hg 0.15 ppm [63]. Moreover, the International Organization for Standardization (ISO) 9917-1 states that As and Pb content in dental water-based cements should be less than 2 and 100 ppm, respectively [64]. The U.S. Food and Drug Administration (FDA) defined the maximum allowed concentration in cosmetics of Hg as 65 ppm and Pb as 10 ppm [65]. However, there is still a need to regulate limits for heavy metal impurities in biomaterials to be used in biomedical applications. In this study, heavy metals were either absent for all cases or, if present, were in undetectable amounts and below the listed limits.

### 2.8. Cell Viability

**Figure 2:**
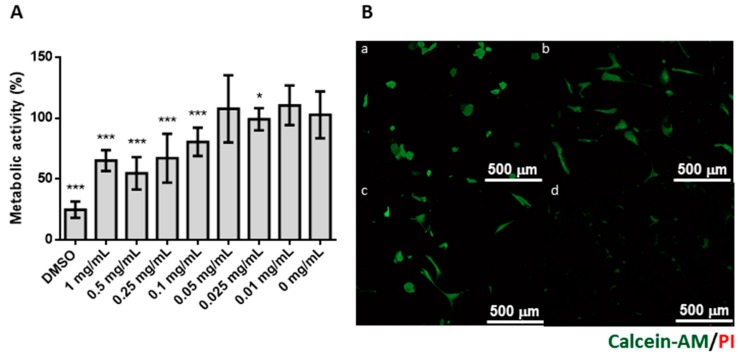
Cytotoxicity evaluation of codfish collagen over MRC-5 human fibroblast cell line. (**A**) Metabolic activity of MRC-5 cell line, measured by MTS assay, when exposed to different concentrations of collagen obtained from codfish skin (0.01–1 mg/mL) in relation to negative control (tissue culture plate). Statistically significant differences were observed: * *p*-value < 0.05 and *** *p*-value < 0.001. (**B**) Live/dead staining with calcein-AM (green) and propidium iodide (red): (a) 0 μg/mm^2^; (b) 0.5 μg/mm^2^; (c) 1.0 μg/mm^2^; and (d) 1.5 μg/mm^2^.

The in vitro cytotoxicity of codfish collagen was investigated using lung fibroblast MRC-5 cells by MTS assay, which is based on the mitochondrial activity of vital cells, reflecting their metabolic activity [66]. Cytotoxicity was calculated comparing each condition with a negative control (Figure 2). The different concentrations of collagen tested showed that in the range of 0.01 to 0.05 mg/mL, collagen does not affect cell metabolism (*p*-value > 0.05). However, at higher concentrations (>0.1 mg/mL), cell metabolism was significantly decreased (*p*-value < 0.001) in relation to the control. Live/dead assay showed that MRC-5 cells remain viable when seeded in collagen coatings, as no dead cells are observed. However, high collagen density may affect cells’ metabolic activity, showed by the decrease of green fluorescence and in accordance with the results from the MTS assay.

### 2.9. Cell Adhesion

**Figure 3:**
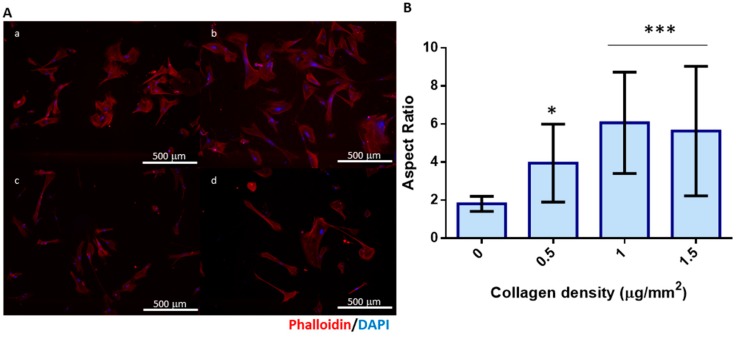
Adhesion of MRC-5 cell line into collagen coatings. Cell morphology was evaluated by (**A**) fluorescence staining of actin–phalloidin (red) and nucleus–DAPI (blue) and (**B**) shape description parameter aspect ratio determined for each condition (a 0 μg/mm^2^ or absence of collagen, the control condition; b 0.5 μg/mm^2^; c 1.0 μg/mm^2^; and d 1.5 μg/mm^2^). Statistically significant differences in respect to the control–absence of collagen–were observed: * *p*-value < 0.05 and *** *p*-value < 0.001.

Cell adhesion and viability of MRC-5 cell lines cultured onto collagen coatings was assessed by fluorescence microscopy (Figure 3). Cells adhered to the collagen coatings and their morphology was affected by the presence of collagen at different densities. The cell aspect ratio increased with the increase of collagen density in the coating. The higher densities of the adhesive protein may allow a higher number of adhesion points, thus resulting in the observed cell elongation and spindle-like morphology, quantitatively translated as higher values of the aspect ratio shape descriptor. This confirms that codfish collagen kept its cell adhesion properties during the extraction processes, indicating that it could be further used in biomedical applications, namely, those considering the development of collagen-based biomaterials to be used as templates for cell culture.

## 3. Materials and Methods

### 3.1. Preparation of Codfish Skin

Codfish skins were removed from cold preserved fish body parts in an industrial plant and kindly provided by Frigoríficos de Ermida, Lda. (Gafanha, Nazaré, Portugal). Frozen skin was thawed at 4 °C for 24 h, with the scales, remaining flesh and bones then manually removed, and the skin cut into small pieces (1.0 cm × 1.0 cm) and washed with ice-cold deionized water. To remove non-collagenous proteins, the skin was treated with 10 volumes of 0.1 M NaOH solution, changed each 24 h, for 72 h at 4 °C under magnetic stirring. The skin was then rinsed with ice-cold deionized water until reaching pH 7. All procedures were carried out at 4 °C to minimize collagen denaturation.

### 3.2. Extraction of Acid-Soluble Collagen from Codfish Skin

All the procedures were carried out at 4 °C and collagen was extracted using an acid-base procedure. The skin was soaked in 0.5 M acetic acid with a sample:solution ratio of 1:10 (*w*/*v*) for 72 h with continuous stirring. The solution was then strained and centrifuged (Eppendorf 5810R, Hamburg, Germany) at 20,000× *g* during 1 h. An accurate volume of 50 mM Tris-HCl containing 2.6 M of NaCl at pH 7.4 buffer was added to the supernatant to reach a final concentration of 0.9 M of NaCl to precipitate collagen. The precipitated collagen was removed from solution by centrifugation at 20,000× *g* for 30 min. A minimal volume of 0.5 M acetic acid was added to the pellet and then dialyzed for 48 h against 10 volumes of 0.1 M acetic acid, 48 h against 0.02 M acetic acid and 48 h against ultrapure water, with the solutions being changed every 24 h. The resulted acid-soluble collagen was freeze-dried for 1 week and stored at −80 °C until further use.

### 3.3. Codfish Extract Purity

Protein content was evaluated by Micro BCA assay (Fisher Scientific, Rockford, IL, USA). Briefly, 1 mg/mL of collagen solution in 20 mM acetic acid was neutralized with 25 mM of Tris-base containing 3% SDS and denatured at 65 °C during 1 h. After equilibration at room temperature the sample was analysed. Sircol assay (Biocolor, County Antrim, UK) was performed according to the manufacturer’s instructions. Collagen type I from rat tail was used as control in Micro BCA and Sircol assays. Extract purity was accessed comparing the protein/collagen content with the initial extract concentration.

### 3.4. Sodium Dozdecyl Sulfate-Polyacrylamide Gel Electrophoresis (SDS-PAGE)

Codfish skin extract was first dissolved at 1 mg/mL in 20 mM acetic acid at room temperature and then mixed with three-fold-concentrated loading buffer containing 0.1 M 1,4-dithiothreitol (DTT) to a final protein mass of 10 μg. Protein samples were heat-denatured at 65 °C for 30 min and analysed by SDS-PAGE according to Laemmli [67] using 4% stacking and 7.5% resolving gels in a Mini Protean III unit (Bio-Rad Laboratories, Hercules, CA, USA) at 27 mA/gel. Protein bands were stained with Coomassie brilliant Blue R250 and destained with 32% (*v*/*v* %) methanol 5.6% (*v*/*v* %) acetic acid solution (destaining solution I), and 5% (*v*/*v* %) methanol and 7% (*v*/*v* %) acetic acid solution (destaining solution II). Rat and bovine collagen were used as controls.

### 3.5. Fourier Transformed Infrared (FTIR) Spectroscopy

Freeze-dried products were individually mixed with vacuum dried potassium bromide (KBr) and pressed into pellets with a hydraulic press. The infrared spectra were obtained in the wavenumber range between 4400 and 400 cm^−1^ at a resolution of 5 cm^−1^, using the infrared spectrometer IRPrestige 21 (Shimadzu, Kyoto, Japan). Each spectrum resulted from the average of 50 scans. Extracted collagen was compared with freeze-dried commercially available rat and bovine collagens.

### 3.6. Protein Conformation

Circular dichroism (CD) spectra of extracts were recorded at 180 to 280 nm on a Jasco J-1500 dichrograph (Jasco Corp., Tokyo, Japan) using a 0.1-cm path length cuvette. Dry collagen was dissolved at 0.1 mg/mL in 5 mM phosphate buffer pH 3. Samples were loaded at 4 °C into precooled CD cuvettes.

### 3.7. Denaturation Temperature

The denaturation temperature of collagen from codfish skin was evaluated by rheology (Kinexus Pro+, Malvern, UK). The viscosity of 1% (*w*/*v*) collagen in 20 mM acetic acid was monitored in the temperature range of 4 °C to 40 °C. Denaturation temperature was determined at 70% and 90% of denatured proteins [68].

### 3.8. Amino Acid Content

The amino acid content was determined by quantitative amino acid analysis using amino acid analyzer Biochrom 30 (Biochrom Ltd., Cambridge, UK) [8]. Briefly, the samples were hydrolyzed and separated by an ion exchange column. After post-column derivatization by ninhydrin, the samples were analyzed at 2 wavelengths: 440 and 570 nm. An internal standard of norleucine was used to determine the concentrations of amino acids in the sample.

### 3.9. Heavy Metals Quantification

The presence of lead (Pb), cadmium (Cd), arsenic (As) and mercury (Hg) in the codfish collagen extracts was quantified from codfish collagen according to Official Methods of analysis of AOAC International, 19th Edition (2012). Collagen was digested with 65% nitric acid and 30% hydrogen peroxide in a microwave digester MWS3+ (Berghof, Münster, Germany). Pb and Cd were quantified by atomic absorption spectrophotometry Perkin Elmer Analyst 600 (Perkin Elmer, Waltham, MA, USA) and As and Hg were quantified using atomic absorption spectrophotometry Perkin Elmer Analyst 600 (Perkin Elmer, Waltham, MA, USA) equipped with a Perkin Elmer FIAS 100 analyzer (Perkin Elmer).

### 3.10. Cell Culture 

Human lung fibroblast cell line MRC-5 was grown as monolayer at 37 °C in DMEM low glucose with phenol red (Sigma-Aldrich, St. Louis, MO, USA) supplemented with sodium bicarbonate, 10% fetal bovine serum and 1% antibiotic/antimycotic. Sub-confluent cells in exponential growth phase were detached with TrypLE™ (Gibco, grand Island, NW, USA) and sub-cultured in fresh medium with appropriate cell density.

### 3.11. MTS Metabolic Activity Assay

Codfish collagen in 20 mM acetic acid with increasing concentration (0–1000 μg/mL) were added to a 48-well plate and evaporated at room temperature in an orbital shaker. Plates were sterilized by 15 min exposure to a 1.2 W UV lamp Omnicure series 2000 EXFO S2000-XLA (Omnicure, Bleichenbach, Germany). MRC-5 cells were seeded at 5 × 10^4^ cells/mL in the collagen containing wells. Wells without collagen or containing 5% DMSO were used as negative and positive controls of cytotoxicity, respectively. Cells were cultured for 24 h at 37 °C in a humidified 5% CO_2_ atmosphere. Metabolic activity was measured using a CellTiter 96^®^ kit (Promega Corporation Madison, USA) according to the manufacturer’s instructions. Absorbance was recorded at 490 nm in a Microplate Reader-Gen 5 2.0 SYNERGY HT (Biotek, Luzern, Switzerland).

### 3.12. Cell Adhesion on Collagen Coatings

Cell adhesion was performed on collagen coatings prepared from solutions with different concentrations. Briefly, collagen in 20 mM acetic acid solution was added to 6-well plates and let to dry overnight at room temperature in an orbital shaker, using the solution concentration and volume needed to achieve different collagen densities (0–1.5 μg/mm^2^). After evaporation at room temperature, collagen was crosslinked with 50 mM EDC/NHS (1:1) solution for 24 h at 4 °C. Coatings were washed 10× with 1× phosphate buffer solution (PBS) and sterilized by UV radiation, as described before. MRC-5 cells were seeded at a density of 5 × 10^4^ cells/mL on coverslips with and without collagen coatings. After 16 h incubation at 37 °C and humidified 5% CO_2_ atmosphere, cell culture medium was removed, cells were washed with PBS and stained. Cell were permeated with 0.2% Triton-X solution for 5 min at room temperature and then incubated with Phalloidin/DAPI in PBS at 250 ng/mL and 1 μg/mL, respectively, for 45 min. 

Live/dead stain was performed with calcein-AM (2 μg/mL) and propidium iodide (1 μg/mL), for 15 min at room temperature. Fluorescent images were acquired in Axio Imager Z1m (Zeiss, Göttingen, Germany), analysed in Zen lite 2.1 (Zeiss, Germany). Shape descriptor parameter aspect ratio was determined using phalloidin signal processing by ImageJ software. Aspect ratio is a shape descriptor corresponding to the ratio between the largest diameter and the smallest diameter perpendicular to it of a round-like particle or body; when the body has nearly a circular shape, the aspect ratio is close to 1, but if the body assumes an elongated shape, for instance caused by stretching, the aspect ratio increases.

### 3.13. Statistical Analysis

All experiments were performed in triplicate and the results are expressed as mean ± standard deviation. Metabolic activity of MRC-5 cells and shape descriptors showed a non-parametric distribution; therefore Mann-Whitney was used to assess statistically significant differences between results. Difference was considered statistically significant at *p* < 0.05.

## 4. Conclusions

The present study showed that collagen type I can be obtained from the skin of codfish with a high degree of purity, representing a valuable strategy for the valorization of a marine by-product. Properties such as molecular weight, amino acid composition and molecular structure were close to those of collagen of mammalian origin. The main difference was in regard to the protein denaturation temperature. Collagen with a low denaturation temperature presents poor gelling properties, which limit its application as a gel-forming agent, causing a processing bottlenecking to achieve cohesive gels at physiological temperatures. However, this could be overcome using chemical crosslinking, such as with 1-ethyl-3-(-3-dimethylaminopropyl) carbodiimide/N-hydroxysuccinimide (EDC/NHS), without significant effects on collagen’s biological functions. Besides the biomechanical properties, collagen is also involved in a wide range of biological functions. Collagen can specifically interact with particular receptors at the cell surface, such as integrins, discoidin-domain receptors, and glycoprotein VI [42], thus signaling cell adhesion, differentiation and growth, as well as cell survival. Moreover, heavy metals were undetectable and therefore below the regulated limits. Marine-origin collagen may be used as a safe source of adhesion points and molecular modulators that can be used in combination with biopolymers for the development of biomaterials with promising biomedical application.

## Figures and Tables

**Table 1:** Amino acid composition of collagen obtained from the skin of codfish, and of rat and bovine commercial collagen (per 1000 residues).

Amino Acid	Rat	Bovine	Codfish
Alanine	111.16	102.04	91.48
Arginine	42.24	32.86	30.45
Aspartic acid	45.32	36.65	38.82
Cystein	0.99	1.24	1.28
Glutamic acid	73.33	59.43	56.08
Glycine	333.18	296.44	266.12
Histidine	3.61	3.11	5.01
Hydroxylysine	9.33	8.86	6.65
Hydroxyproline	96.06	78.35	39.60
Isoleucine	7.48	6.74	5.61
Leucine	23.29	17.50	16.51
Lysine	27.07	22.20	19.62
Methionine	8.03	7.81	15.04
*N*-isobutylglycine	12.70	14.33	13.75
Phenylalanine	14.62	11.58	12.70
Proline	109.21	89.89	62.69
Serine	42.74	32.03	53.87
Threonine	18.79	13.20	16.89
Tyrosine	3.76	1.48	2.25
Valinine	17.08	12.86	12.02

**Table 2:** Denaturing temperature (10%) and temperature until which collagen is considered in native structure (30%).

Collagen Origin	Temperature According to % of Native Collagen	
	30	10
Codfish	15.77 ± 0.09	18.24 ± 0.40
Rat	39.65 ± 0.02	40.08 ± 0.01
Bovine	33.19 ± 0.02	35.34 ± 0.03

## References

[1] Ricard-Blum S. (2011). The collagen family. Cold Spring Harb. Perspect. Biol..

[2] Lee C.H., Singla A., Lee Y. (2001). Biomedical applications of collagen. Int. J. Pharm..

[3] Cen L., Liu W., Cui L., Zhang W., Cao Y. (2008). Collagen tissue engineering: Development of novel biomaterials and applications. Pediat. Res..

[4] Maeda M., Tani S., Sano A., Fuijoka K. (1999). Microstructure and release characteristics of the minipellet, a collagen based drug delivery system for controlled release of protein drugs. J. Control. Release.

[5] Albu M.G., Titorencu I., Ghica M., Pignatello R. (2011). Collagen-based drug delivery systems for tissue engineering. Biomaterials Applications for Nanomedicine.

[6] Pati F., Datta P., Adhikari B., Dhara S., Ghosh K., Mohapatra P.K.D. (2012). Collagen scaffolds derived from fresh water fish origin and their biocompatibility. J. Biomed. Mater. Res. Part A.

[7] Hayashi Y., Yanagiguchi K., Yamada S. (2015). Fish collagen as a scaffold. Austin J. Biomed. Eng..

[8] Barros A.A., Aroso I.M., Silva T.H., Mano J.F., Duarte A.R.C., Reis R.L. (2015). Water and carbon dioxide: Green solvents for the extraction of collagen/gelatin from marine sponges. ACS Sustain. Chem. Eng..

[9] Silva T.H., Moreira-Silva J., Marques A.L.P., Domingues A., Bayon Y., Reis R.L. (2014). Marine origin collagens and its potential applications. Mar. Drugs.

[10] Silva J.C., Barros A.A., Aroso I.M., Fassini D., Silva T.H., Reis R.L., Duarte A.R.C. (2016). Extraction of collagen/gelatin from the marine demosponge chondrosia reniformis (nardo, 1847) using water acidified with carbon dioxide—Process optimization. Ind. Eng. Chem. Res..

[11] Pozzolini M., Bruzzone F., Berilli V., Mussino F., Cerrano C., Benatti U., Giovine M. (2012). Molecular characterization of a nonfibrillar collagen from the marine sponge chondrosia reniformis nardo 1847 and positive effects of soluble silicates on its expression. Mar. Biotechnol..

[12] Pozzolini M., Scarfi S., Gallus L., Castellano M., Vicini S., Cortese K., Gagliani M.C., Bertolino M., Costa G., Giovine M. (2018). Production, characterization and biocompatibility evaluation of collagen membranes derived from marine sponge chondrosia reniformis nardo, 1847. Mar. Drugs.

[13] Pozzolini M., Scarfi S., Mussino F., Ferrando S., Gallus L., Giovine M. (2015). Molecular cloning, characterization, and expression analysis of a prolyl 4-hydroxylase from the marine sponge chondrosia reniformis. Mar. Biotechnol..

[14] Tziveleka L.A., Ioannou E., Tsiourvas D., Berillis P., Foufa E., Roussis V. (2017). Collagen from the marine sponges axinella cannabina and suberites carnosus: Isolation and morphological, biochemical, and biophysical characterization. Mar. Drugs.

[15] Ehrlich H., Wysokowski M., Zoltowska-Aksamitowska S., Petrenko I., Jesionowski T. (2018). Collagens of poriferan origin. Mar. Drugs.

[16] Cheng X.C., Shao Z.Y., Li C.B., Yu L.J., Raja M.A., Liu C.G. (2017). Isolation, characterization and evaluation of collagen from jellyfish rhopilema esculentum kishinouye for use in hemostatic applications. PLoS ONE.

[17] Widdowson J.P., Picton A.J., Vince V., Wright C.J., Mearns-Spragg A. (2018). In vivo comparison of jellyfish and bovine collagen sponges as prototype medical devices. J. Biomed. Mater. Res. Part B Appl. Biomater..

[18] Sewing J., Klinger M., Notbohm H. (2017). Jellyfish collagen matrices conserve the chondrogenic phenotype in two- and three-dimensional collagen matrices. J. Tissue Eng. Regen. Med..

[19] Exposito J.Y., Larroux C., Cluzel C., Valcourt U., Lethias C., Degnan B.M. (2008). Demosponge and sea anemone fibrillar collagen diversity reveals the early emergence of a/c clades and the maintenance of the modular structure of type v/xi collagens from sponge to human. J. Biol. Chem..

[20] Nowack H., Nordwig A. (1974). Sea-anemone collagen—Isolation and characterization of cyanogen-bromide peptides. Eur. J. Biochem..

[21] Benayahu D., Sharabi M., Pomeraniec L., Awad L., Haj-Ali R., Benayahu Y. (2018). Unique collagen fibers for biomedical applications. Mar. Drugs.

[22] Cozza N., Bonani W., Motta A., Migliaresi C. (2016). Evaluation of alternative sources of collagen fractions from loligo vulgaris squid mantle. Int. J. Biol. Macromol..

[23] Coelho R.C.G., Marques A.L.P., Oliveira S.M., Diogo G.S., Pirraco R.P., Moreira-Silva J., Xavier J.C., Reis R.L., Silva T.H., Mano J.F. (2017). Extraction and characterization of collagen from antarctic and sub-antarctic squid and its potential application in hybrid scaffolds for tissue engineering. Mater. Sci. Eng. C.

[24] Dai M.L., Liu X., Wang N.P., Sun J. (2018). Squid type ii collagen as a novel biomaterial: Isolation, characterization, immunogenicity and relieving effect on degenerative osteoarthritis via inhibiting stat1 signaling in pro-inflammatory macrophages. Mater. Sci. Eng. C-Mater. Biol. Appl..

[25] Ferrario C., Leggio L., Leone R., Di Benedetto C., Guidetti L., Cocce V., Ascagni M., Bonasoro F., La Porta C.A.M., Carnevali M.D.C. (2017). Marine-derived collagen biomaterials from echinoderm connective tissues. Mar. Environ. Res..

[26] Ovaska M., Bertalan Z., Miksic A., Sugni M., Di Benedetto C., Ferrario C., Leggio L., Guidetti L., Alava M.J., La Porta C.A.M. (2017). Deformation and fracture of echinoderm collagen networks. J. Mech. Behav. Biomed. Mater..

[27] Wilkie I.C., Emson R.H., Young C.M. (1993). Smart collagen in sea lilies. Nature.

[28] Bao Z.X., Sun Y., Rai K., Peng X.Y., Wang S.L., Nian R., Xian M. (2018). The promising indicators of the thermal and mechanical properties of collagen from bass and tilapia: Synergistic effects of hydroxyproline and cysteine. Biomater. Sci..

[29] Fassini D., Duarte A.R.C., Reis R.L., Silva T.H. (2017). Bioinspiring chondrosia reniformis (nardo, 1847) collagen-based hydrogel: A new extraction method to obtain a sticky and self-healing collagenous material. Mar. Drugs.

[30] Bernhardt A., Paul B., Gelinsky M. (2018). Biphasic scaffolds from marine collagens for regeneration of osteochondral defects. Mar. Drugs.

[31] Fernandes-Silva S., Moreira-Silva J., Silva T.H., Perez-Martin R.I., Sotelo C.G., Mano J.F., Duarte A.R.C., Reis R.L. (2013). Porous hydrogels from shark skin collagen crosslinked under dense carbon dioxide atmosphere. Macromol. Biosci..

[32] Gómez-Guillén M.C., Turnay J., Fernández-Díaz M.D., Ulmo N., Lizarbe M.A., Montero P. (2002). Structural and physical properties of gelatin extracted from different marine species: A comparative study. Food Hydrocoll..

[33] Wang Y., Regenstein J.M. (2009). Effect of edta, hcl, and citric acid on ca salt removal from asian (silver) carp scales prior to gelatin extraction. J. Food Sci..

[34] Kittiphattanabawon P., Benjakul S., Visessanguan W., Shahidi F. (2010). Isolation and characterization of collagen from the cartilages of brownbanded bamboo shark (chiloscyllium punctatum) and blacktip shark (carcharhinus limbatus). LWT Food Sci. Technol..

[35] Matmaroh K., Benjakul S., Prodpran T., Encarnacion A.B., Kishimura H. (2011). Characteristics of acid soluble collagen and pepsin soluble collagen from scale of spotted golden goatfish (parupeneus heptacanthus). Food Chem..

[36] Fengxiang Z., Anning W., Zhihua L., Shengwen H., Lijun S. (2011). Preparation and characterisation of collagen from freshwater fish scales. Food Nutr. Sci..

[37] Jeong H.S., Venkatesan J., Kim S.K. (2013). Isolation and characterization of collagen from marine fish (thunnus obesus). Biotechnol. Bioprocess Eng..

[38] Muralidharan N., Jeya Shakila R., Sukumar D., Jeyasekaran G. (2013). Skin, bone and muscle collagen extraction from the trash fish, leather jacket (odonus niger) and their characterization. J. Food Sci. Technol..

[39] El-Rashidy A.A., Gad A., Abu-Hussein A.E.H.G., Habib S.I., Badr N.A., Hashem A.A. (2015). Chemical and biological evaluation of egyptian nile tilapia (oreochromis niloticas) fish scale collagen. Int. J. Biol. Macromol..

[40] Kozlowska J., Sionkowska A., Skopinska-Wisniewska J., Piechowicz K. (2015). Northern pike (esox lucius) collagen: Extraction, characterization and potential application. Int. J. Biol. Macromol..

[41] Huang C.Y., Kuo J.M., Wu S.J., Tsai H.T. (2016). Isolation and characterization of fish scale collagen from tilapia (oreochromis sp.) by a novel extrusion–hydro-extraction process. Food Chem..

[42] International A. (2008). Standard guide for characterization of type i collagen as starting material for surgical implants and substrates for tissue engineered medical products (temps). F 2212-08.

[43] Gelse K., Pöschl E., Aigner T. (2003). Collagens—Structure, function, and biosynthesis. Adv. Drug Deliv. Rev..

[44] Skierka E., Sadowska M. (2007). The influence of different acids and pepsin on the extractability of collagen from the skin of baltic cod (gadus morhua). Food Chem..

[45] Żelechowska E., Sadowska M., Turk M. (2010). Isolation and some properties of collagen from the backbone of baltic cod (gadus morhua). Food Hydrocoll..

[46] Kimura S., Ohno Y. (1987). Fish type i collagen: Tissue-specific existence of two molecular forms, (α1)2α2 and α1α2α3, in alaska pollack. Comp. Biochem. Physiol. Part B Comp. Biochem..

[47] Matsui R., Ishida M., Kimura S. (1991). Characterization of an α3 chain from the skin type i collagen of chum salmon (oncoorhynchus keta). Comp. Biochem. Physiol. Part B Comp. Biochem..

[48] Doyle B.B., Bendit E.G., Blout E.R. (1975). Infrared spectroscopy of collagen and collagen-like polypeptides. Biopolymers.

[49] Singh P., Benjakul S., Maqsood S., Kishimura H. (2011). Isolation and characterisation of collagen extracted from the skin of striped catfish (pangasianodon hypophthalmus). Food Chem..

[50] Kiew P.L., Don M.M. (2013). The influence of acetic acid concentration on the extractability of collagen from the skin of hybrid clarias sp. And its physicochemical properties: A preliminary study. Focus. Mod. Food Ind..

[51] Plepis A.M.G., Goissis G., Gupta D.K.D. (1996). Dielectric and pyroelectric characterization of anionic and native collagen. Polym. Eng. Sci..

[52] Veeruraj A., Arumugam M., Ajithkumar T., Balasubramanian T. (2012). Isolation and characterization of drug delivering potential of type-i collagen from eel fish evenchelys macrura. J. Mater. Sci. Mater. Med..

[53] Friess W., Lee G. (1996). Basic thermoanalytical studies of insoluble collagen matrices. Biomaterials.

[54] Pati F., Adhikari B., Dhara S. (2010). Isolation and characterization of fish scale collagen of higher thermal stability. Bioresour. Technol..

[55] Duan R., Zhang J., Du X., Yao X., Konno K. (2009). Properties of collagen from skin, scale and bone of carp (cyprinus carpio). Food Chem..

[56] Benjakul S., Nalinanon S., Shahidi F., Simpson B.K. (2012). Fish collagen. Food Biochemistry and Food Processing.

[57] Piez K.A., Gross J. (1960). Amino acid composition of some fish collagens—Relation between composition and structure. J. Biol. Chem..

[58] Ramachan G.N., Bansal M., Bhatnaga R.S. (1973). Hypothesis on role of hydroxyproline in stabilizing collagen structure. Biochim. Biophys. Acta.

[59] Persikov A.V., Ramshaw J.A.M., Kirkpatrick A., Brodsky B. (2005). Electrostatic interactions involving lysine make major contributions to collagen triple-helix stability. Biochemistry.

[60] Fallas J.A., Gauba V., Hartgerink J.D. (2009). Solution structure of an abc collagen heterotrimer reveals a single-register helix stabilized by electrostatic interactions. J. Biol. Chem..

[61] Piez K.A. (1984). Extracellular Matrix Biochemistry.

[62] Gauza-Włodarczyk M., Kubisz L., Włodarczyk D. (2017). Amino acid composition in determination of collagen origin and assessment of physical factors effects. Int. J. Biol. Macromol..

[63] (2012). Usp chapter <232>, elemental impurities—Limits. Second Supplement to USP 35—NF 30.

[64] International Organization for Standardization (2007). Dentistry: Water-Basedcements—Part 1: Powder/Liquid Acid-Base Cements.

[65] U.S. Food and Drug Administration (2016). Draft Guidance for Industry: Lead in Cosmetic Lip Products and Externally Applied Cosmetics: Recommended Maximum Level.

[66] Wang P., Henning S.M., Heber D. (2010). Limitations of mtt and mts-based assays for measurement of antiproliferative activity of green tea polyphenols. PLoS ONE.

[67] Laemmli U.K. (1970). Cleavage of structural proteins during the assembly of the head of bacteriophage t4. Nature.

[68] Lepock J.R. (2005). Measurement of protein stability and protein denaturation in cells using differential scanning calorimetry. Methods.

